# Impact of Exogenous Melatonin Application on Photosynthetic Machinery under Abiotic Stress Conditions

**DOI:** 10.3390/plants12162948

**Published:** 2023-08-15

**Authors:** Sameera Karumannil, Tanveer Alam Khan, Sajeesh Kappachery, Mayank Anand Gururani

**Affiliations:** Biology Department, College of Science, UAE University, Al Ain P.O. Box 15551, United Arab Emirates

**Keywords:** melatonin, exogenous, abiotic stress, photosynthesis, antioxidant

## Abstract

Inhospitable conditions that hinder plant growth and development encompass a range of abiotic stresses, such as drought, extreme temperatures (both low and high), salinity, exposure to heavy metals, and irradiation. The cumulative impact of these stresses leads to a considerable reduction in agricultural productivity worldwide. The generation of reactive oxygen species (ROS) is a shared mechanism of toxicity induced by all these abiotic stimuli in plants, resulting in oxidative damage and membrane instability. Extensive research has shed light on the dual role of melatonin in plants, where it serves as both a growth regulator, fostering growth and development, and a potent protector against abiotic stresses. The inherent potential of melatonin to function as a natural antioxidant positions it as a promising biostimulant for agricultural use, bolstering plants’ abilities to withstand a wide array of environmental challenges. Beyond its antioxidant properties, melatonin has demonstrated its capacity to regulate the expression of genes associated with the photosynthetic process. This additional characteristic enhances its appeal as a versatile chemical agent that can be exogenously applied to plants, particularly in adverse conditions, to improve their resilience and optimize photosynthetic efficiency in every phase of the plant life cycle. An examination of the molecular mechanisms underlying the stress-protective effects of exogenous melatonin on the photosynthetic machinery of plants under various abiotic stresses is presented in this paper. In addition, future prospects are discussed for developing stress-tolerant crops for sustainable agriculture in challenging environments.

## 1. Introduction

**Figure 1:**
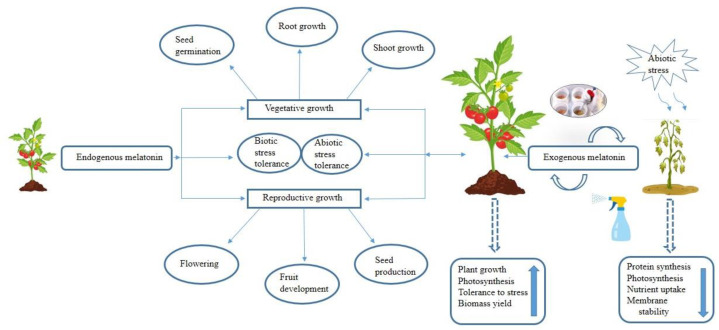
Melatonin plays a pivotal role in the growth and development of plants, exerting influence on both vegetative (e.g.,: seed germination, root growth, and shoot growth) and reproductive (e.g.,: flowering, fruit development, and seed production) stages. The application of exogenous melatonin or the enhanced endogenous production in response to abiotic stress conditions has been shown to improve physiological parameters of crops including growth, maintaining photosynthesis efficiency, stress tolerance, increasing biomass yield, and influencing protein synthesis and overall plant metabolism.

N-acetyl-5-methoxytryptamine, commonly known as melatonin, is a ubiquitous and natural biomolecule that plays crucial roles in various cellular and physiological processes across animals and plants [1]. Melatonin is present in several plant families including monocots and dicots exhibiting notable variations dependent on the species, growth and developmental stages, and tissue types [2]. The constitutive presence of melatonin in diverse plant organs (e.g., roots, stems, leaves, fruits, flowers, and seeds) suggests its substantial contribution to plant growth, maturation, and resilience impacting multiple physiological processes [3]. Melatonin plays a pivotal role in seed germination [4], lateral root development [5], photosynthesis [6], leaf senescence [7], flowering and fruit set [1], and osmoregulation [8] (Figure 1).

Melatonin synthesis occurs in chloroplast and mitochondria through two primary pathways that rely on tryptophan as a precursor. The N-acetylserotonin pathway is predominantly associated with normal growth conditions with the final step in the chloroplast, while the serotonin/methoxytryptamine pathway is primarily linked to the senescence process and its final step occurs in the cytosol [9]. It plays a crucial role in protecting the photosynthetic apparatus and stomatal systems, and the regulation of various enzymes involved in amino acid, carbohydrate, lipid, nitrogen, sulfur, and phosphorus metabolism (Figure 1). In secondary metabolism, melatonin acts as a catalyst to produce photoprotective compounds such as carotenoids, anthocyanins, and flavonoids (Figure 1). Moreover, melatonin also regulates the synthesis of diverse plant hormones, including auxin, cytokinins, abscisic acid, gibberellins, ethylene, and jasmonate [10]. Furthermore, the remarkable ability of melatonin to regulate both enzymatic and non-enzymatic antioxidant activity in stressful conditions is considered one of its most significant functions in living organisms [11]. By scavenging reactive oxygen species (ROS) and nitrogen species (RNS), melatonin improves the cellular antioxidant capacity playing a role in cell protection and tissue maintenance in relation to oxidative stress [12,13,14]. 

Over the past decade, there has been a focus on mitigating the impacts of environmental stress through the application of appropriate fertilizers, soil enhancement, and various irrigation strategies. In parallel, alternative approaches involving the application of various external agents during plant growth have been tested, notably, applications employing melatonin may effectively enhance stress resistance in the plant, particularly by protecting the photosynthetic apparatus [15]. It is universally acknowledged that exogenous melatonin application positively alters the growth and development of plants under abiotic stress by (a) reducing oxidative stress activating the antioxidant system, (b) changing the expression of stress-responsive genes and heat shock proteins (HSPs), (c) increasing the accumulation of osmolytes, secondary metabolites, and amino acids, (d) modulating the biosynthesis of endogenous melatonin and other growth regulators, (e) increasing the expression of photosynthesis-related proteins, (f) upregulating the expression of chlorophyll-synthesis-related genes, and (g) inhibiting the expression of chlorophyll degradation-associated genes [16,17,18,19,20]. This review aims to provide an overview of recent advancements in understanding the protective effects of melatonin application on photosynthetic machinery, with a particular focus on mitigating the adverse impacts of drought, salinity, temperature, irradiance, and metal toxicity.

## 2. Impact of Abiotic Stress on Photosynthetic Components

Photosynthesis is the fundamental biochemical process in green plant tissues that sustains the biosphere and serves as the basis for the food chain. Although photosynthesis is essential for plant growth and development, it is highly sensitive to environmental stresses. The abiotic stressors, including drought, temperaturevariations, salinity, heavy metal contamination, and UV radiation, pose a severe risk to plant growth and development, hinder agricultural productivity, and threaten the overall ecosystem [21,22,23,24]. These factors have deleterious effects on chlorophyll biosynthesis, photosystem functionality, electron transport mechanisms, gas exchange parameters, chloroplast shape, and many other processes that essentially decrease the photosynthetic efficiency of plants [25,26,27,28,29].

Numerous studies have evidenced that stomatal closure is the plant’s primary protective mechanism in response to drought stress. However, this response leads to a decrease in net CO_2_ assimilation and ultimately impacts photosynthesis [30,31]. Additionally, extreme drought conditions can further impair photosynthesis by limiting the activity of the ribulose-1,5-bisphosphate carboxylase/oxygenase (RuBisCO) enzyme due to the prevention of its regeneration leading to severe inhibition of CO_2_ assimilation in the photosynthetic process and PSII reaction center [32]. Furthermore, studies conducted by Ye et al. [33] indicate that drought stress disrupts the balance of ROS, which therefore causes thylakoid peroxidation, chlorophyll degradation, and decreased photosynthetic rate in plants.

Chloroplasts, the organelle responsible for photosynthesis, are highly susceptible to damage in response to salt stress. The increasing salt concentration leads to membrane disruption, blurring of the borders between grana and stroma lamellae, disorganization of the thylakoid arrangement, and even disintegration of chloroplasts [34]. Furthermore, the salt stress causes the accumulation of sodium (Na^+^) and chloride (Cl^-^) ions within the chloroplast, leading to a decrease in plant water potential, inhibition of photosynthesis, increase in ROS production, and disruptions in electron transport [35]. Additionally, salt stress also has other adverse effects on photosynthesis parameters reducing the levels of metabolites and pigments and inhibiting several physiological and cellular processes [36,37].

Photosynthesis is one of the most temperature-sensitive cell functions in plants. Both high- and low-temperature stresses present detrimental effects on photosynthetic efficiency, leading to a decrease in the levels of photosynthetic pigments in plant leaves [38,39]. Heat stress primarily affects the carbon metabolism in the stroma and photochemical reactions in thylakoid lamellae of the chloroplast [40]. Under heat stress conditions, chloroplasts undergo significant changes, such as altered structural organization of thylakoids, loss of grana stacking, and grana swelling [41]. Furthermore, the activity of photosystem II (PSII) is drastically decreased or even halted during extremely high-temperature conditions. The reduction in PSII activity results in electron leakage from the thylakoid membrane, leading to the production of ROS [42]. High-temperature stress also primarily affects key proteins of the photosynthesis process, including RuBisCO, cytochrome b559, and plastoquinone. Similarly, cold stress disrupts various aspects of plant photosynthesis. According to research by Hou et al. [43], chilling stress induces significant changes in the physiological, molecular, and metabolic activity of plants as well as alterations in membrane permeability and antioxidant activity. Cold stress directly impacts the photosynthetic system, particularly by causing photoinhibition at both PSI and PSII and a decline in CO_2_ assimilation [31,44]. Furthermore, chilling stress also impairs stomatal conductance, transpiration rate, carbon reduction cycle, thylakoid membrane, and electron transport [31,43], resulting in a decrease in the overall photosynthesis activity of crops.

Both anthropogenic and natural sources of heavy metal stress have a detrimental impact on plant life. Studies have demonstrated the negative effect of cadmium poisoning in the reduction in the size and number of chloroplasts, degradation of chlorophyll, and increase in plastoglobuli accumulation [45]. Exposure to chromium has been reported to damage chloroplast ultrastructure, decrease chlorophyll content and impair magnesium and nitrogen absorption [46]. Furthermore, lead toxicity increases the activity of chlorophyllase, and alters the composition of lipids in thylakoid membranes [47]. Lead also negatively affects CO_2_ fixation by reducing stomatal conductance, inducing oxidative stress, and impairing the activity of RuBisCO [47]. Furthermore, nickel stress alters the structure of the lipid membrane and interferes with the functions of chlorophylls and RuBisCO [48].

Although light plays a crucial role in plant development, excessive or insufficient quantities can induce stress on plants. Extremely low light levels hinder plant growth due to insufficient energy, while excessively high light levels can lead to photoinhibition as the light-harvesting system becomes overwhelmed [49]. Under low light stress, plants experience a reduction in net photosynthetic rate, stomatal conductance, transpiration rate, the quantum efficiency of PSII, and water use efficiency [50]. High light intensity can cause damage to both PSI and PSII, resulting in decreased photochemical efficiency and mitochondrial activity [51]. This process is often accompanied by an emission of dissipated energy in the form of heat or fluorescence. Prolonged periods of acute light stress, particularly when combined with high temperatures, can lead to the photooxidation of chlorophyll and triggers the activity of an isozyme of Chl-b reductase [52].

**Figure 2:**
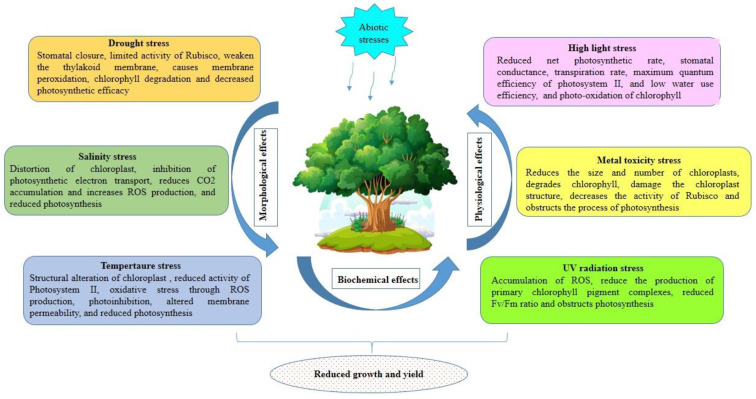
Overview of the impact of abiotic stress on photosynthetic components of crop plants. Drought, salinity, temperature, high light, metal toxicity, and UV radiation impair photosynthesis parameters leading to biochemical, physiological, and morphological changes, which impact plant growth and development.

Increased UV radiation poses a significant threat to all living organisms, but photosynthetic species are particularly vulnerable due to their reliance on sunlight to survive. Plants exposed to UV radiation exhibit reduced production of primary chlorophyll pigments [53]. UV-B radiation can cause DNA damage and accumulation of reactive oxygen species [54]. Furthermore, prolonged exposure progressively diminishes the maximum quantum yield of photosystem II (Fv/Fm), indicating a decline in the efficiency of photosynthesis [55]. Figure 2 depicts the general effects of abiotic stresses on the photosynthetic components of plants.

## 3. Melatonin and Its Protective Effects on Photosynthetic Machinery under Various Abiotic Stresses

Melatonin has emerged as a crucial plant master-regulator due to its ability to manage oxidative stress from generating free radicals during photosynthesis and respiration. Additionally, melatonin functions as a potent plant biostimulant against biotic and abiotic challenges due to promoting (a) the accumulation of soluble sugars and proline levels, (b) the modulation of the activity of different antioxidant enzymes that scavenge free radicals such as superoxide dismutase (SOD), catalase (CAT), peroxidase (POD), ascorbate peroxidase (APX), glutathione peroxidase (GPX), glutathione reductase (GR), dehydroascorbate reductase (DHAR), and monodehydroascorbate reductase (MDHAR) [56,57], and (c) the upregulation of genes associated to stress tolerance [46,58].

Recent research results have suggested that applying exogenous melatonin can improve plant photosynthetic rates. Melatonin has been shown to inhibit chlorophyll degradation, increase leaf photosynthetic rates by enhancing RuBisCO activity, and promote the accumulation of dry matter in plants [59]. According to Ren et al. [5], exogenous melatonin application on stressed plants resulted in a remarkable enhancement in biomass, leaf area, and photosynthetic efficiency. Many studies have reported that exogenous melatonin further enhances plant tolerance in response to drought stress [60,61,62]. Despite the role of melatonin in the stress mitigation response which has been reported in many plant species, there is still limited understanding regarding its regulatory mechanism on photosynthesis and its protective effect on the photosynthetic machinery. In this context, we have outlined the protective effects of melatonin on the photosynthetic machinery of plants exposed to a range of abiotic stresses. Melatonin’s multifaceted impact on photosynthesis offers valuable insights into its potential as a key regulator in promoting plant resilience and survival under adverse conditions.

### 3.1. Drought

Among the abiotic stresses, drought stress poses a major threat to plant growth, survival, and productivity. When faced with this challenge in environmental conditions, plants trigger diverse physiological, biochemical, and molecular responses to aid in their adaptation and survival [63]. One component in this adaptation process is the plant growth regulator, melatonin. Research has shown that melatonin plays a crucial role in the regulation of photosynthetic processes and bolsters the antioxidant defense system in plants when subjected to drought stress [64]. Moreover, melatonin contributes to enhanced stomatal conductance and improved photosynthetic efficiency protecting against chlorophyll degradation during drought [65].

In wheat, melatonin has proven to be a valuable component in mitigating the negative impacts of drought stress. Melatonin treatments led to a reduction in ROS and membrane damage, while simultaneously enhancing antioxidant activity [66]. The effects extend to the improvement in various aspects of leaf structure, including large epidermal cells and undamaged chloroplast granules resulting in enhanced photosynthetic activity [66]. Notably, melatonin’s application downregulates the expression of the gene *pheophorbide A oxygenase (PAO)*, a key component of chlorophyll degradation under stressful circumstances. Furthermore, melatonin positively influences the expression of key photosynthetic genes (e.g., *RBCS2*), leading to increased overall photosynthetic efficiency and supporting crop growth even in drought-stressed environments [67]. Similar positive outcomes were observed in oats subjected to PEG-induced drought stress when melatonin was applied [68]. The treatment improved performance indices, such as PI_ABS_ and PI_total_, and showed a remarkable impact on quantum yield and efficiency of PSII. Moreover, the application of melatonin led to the upregulation of five genes (*PsbA, PsbB, PsbC, PsbD*, and *PsbO*) encoding core proteins of PSII, further supporting the enhancement of photosynthesis performance and resilience [69].

Studies focusing on maize seedlings have revealed the impact of melatonin in mitigating the detrimental effects of drought on plant growth. Upon melatonin treatment, several favorable changes were observed. Firstly, stomatal conductance was improved, leading to the better regulation of water loss and helping plants against drought stress [61,69]. Secondly, photosynthetic efficiency was enhanced, contributing to the overall growth of the maize seedling. Thirdly, the treatment led to a reduction in electrolyte leakage and the accumulation of H_2_O_2_. Finally, the beneficial effects of melatonin treatment extend to the accumulation of osmoprotectants, stabilization of chloroplast structure, and supported cell expansion [61,62,69].

The endogenous concentration of melatonin showed a significant correlation with increased photosynthetic capability and stress-related phytohormones when exposed to water scarcity conditions [70]. Exogenous melatonin treatments further amplified this effect by increasing the levels of indole acetic acid (IAA) and zeatin, while reducing the synthesis of the stress-related molecules H_2_O_2_ and aminocyclopropane-1-carboxylic acid (ACC) [70]. Furthermore, supplementation with melatonin has been found to counteract drought stress effects by modulating the abscisic acid (ABA) levels. It has been reported that melatonin treatment upregulates genes involved in ABA catabolism, and simultaneously suppresses genes related to ABA biosynthesis, ultimately alleviating stomatal closure under drought conditions [57,70]. The dual action resulted in improved photosynthetic capacity and water usage efficiency in wheat [70].

The exogenous application of melatonin in various other crops has shown promising results in enhancing drought resistance. According to Campos et al. [63], the application of lower concentrations in coffee expands the root system and protects the photosynthetic apparatus, improving gas exchange, carboxylation efficiency, and chlorophyll contents. Similarly, the application in *Triticale hexaploide* increased stomatal conductance, net photosynthetic rate, transpiration rate, and relative chlorophyll concentration while enhancing the activities of SOD and POD, and reducing ROS and malonaldehyde (MDA) content [71]. In kiwi plants, melatonin promoted electron transport in PSII, prevented stomatal closure, and increased light energy absorption through the induction of the transcription of 11 genes encoding for CO_2_ fixation enzymes, mitigating the suppression of biomass accumulation and photosynthesis impairment caused by drought [64]. Melatonin treatment through foliar and root irrigation in soybean resulted in a slight increase in chlorophyll levels and a reduction in ABA content compared with control plants, indicating improved drought resistance [72]. Furthermore, in *Chrysanthemum* seedlings, exogenous application lessened the impact of drought stress on the photosynthetic mechanism, promoted seedling growth, reduced water loss, preserved chlorophyll levels, increased soluble protein and sugar levels, and enhanced antioxidant enzyme activity, leading to improved drought tolerance [73]. Finally, foliar treatment on *Moringa oleifera* enhanced photosynthetic pigment constituents, and the accumulation of IAA and phenolics, resulting in increased photosynthesis [74]. *Ranunculus asiaticus* and *Phoebe* showed increased accumulation of photosynthetic pigments [75,76] following melatonin treatment [75,76].

Table 1 summarizes recent reports of exogenous melatonin application on various crops and its impact on the photosynthetic machinery under drought stress conditions. Overall, these studies suggest that exogenous melatonin increases antioxidant activity, photosynthetic performance, and proline accumulation, providing drought resistance and serving as a stress reliever. Furthermore, it effectively reduces the accumulation of ABA- and ROS-induced dryness.

### 3.2. Salinity

Salinity stress poses a significant challenge to crop productivity, affecting critical physiological and biochemical processes such as photosynthesis. The adverse effects include stomatal closure and disruptions in photosynthetic parameters. Various studies have demonstrated that melatonin has shown a promising role in alleviating these effects in plants by increasing photosynthetic pigment content and strengthening antioxidant defenses.

Melatonin has shown remarkable effectiveness in mitigating the negative impact of salinity stress on cucumber plants. It effectively reduces the decline in the net photosynthetic rate, the maximum quantum efficiency of PSII, and the overall chlorophyll content during salinity stress. Additionally, melatonin treatment lowers electrolyte leakage and MDA concentration and increases chlorophyll production in leaves under salinity stress [77]. In tomato plants subjected to NaCl stress, exogenous melatonin treatment exhibits a reduction in chlorophyll degradation, regulation of the distribution of photosynthetic electrons to minimize the generation of ROS, and enhancement of the activity of enzymes involved in the ascorbate–glutathione cycle [78]. This treatment improved tomato seedling growth under NaCl by increasing antioxidant enzyme activity, proline content, and glycine betaine levels, while decreasing glycolate oxidase activity, chlorophyll degradation, and ROS levels [79]. Furthermore, Zhou et al. [80] have reported that melatonin pretreatment in tomato saline conditions led to an increase in Fv/Fm, the photochemical quenching coefficient, and the percentage of open PSII centers. Exogenous melatonin supplementation has also been shown to enhance sugar accumulation, chlorophyll production, PS-II protection, and upregulation of genes associated with antioxidant protection under salt-stressed conditions in *Beta vulgaris*, *Phaseolus vulgaris*, and rice [81,82,83]. In cotton seedlings exposed to salt stress, melatonin effectively prevents the production of ROS, resulting in increased biomass and chlorophyll content, and controls the photosynthetic properties of the plants. It also promoted control over the photosynthesis properties through stomatal opening and the preservation of the mitochondria and grana lamella structure of cotton chloroplasts during salt stress [84].

In wheat seedlings, exogenous melatonin pre-treatment partially mitigated salt-induced suppression of plant growth, as evidenced by improvements in shoot dry weight, IAA concentration, leaf photosynthesis rate, maximum photochemistry efficiency of PSII, and chlorophyll content [85]. Similarly, the application of melatonin in salt-stressed seeds and seedlings regulated the levels of soluble protein, sugar and Ca^2+^, ion compartmentation in roots and leaves, and changes in amino acid contents resulting in maintained high water status and a low level of H_2_O_2_, ultimately leading to a high photosynthetic rate [86]. In oats, melatonin has been found to increase PSII efficiency under salt stress by reducing stress-related PSII damage and encouraging enzymatic antioxidants to scavenge ROS. Melatonin-treated oats showed higher chlorophyll content and proline accumulation, along with reduced levels of MDA and inhibition of electrolyte leakage [13]. Application of exogenous melatonin also significantly improved sugar beet tolerance to salt stress, as evidenced by the higher levels of net photosynthetic rate, chlorophyll fluorescence, and chlorophyll content compared to untreated salt-stressed plants [81].

Table 2 summarizes the physiological and biochemical alterations in the photosynthetic apparatus followed by the application of melatonin and its impact on photosynthesis in different crops under salinity stress conditions.

### 3.3. Temperature

Temperature stress poses a significant threat to plant growth and development. Photosynthesis acts as an important sensor of temperature stress by often being inhibited before other cellular processes are affected. To counteract the adverse impact of this stress, the application of melatonin emerges as a very promising strategy [87,88]. Melatonin has demonstrated significant effects on the production of total phenols and flavonoids, contributing to the enhancement of heat stress tolerance in grapes [89]. The modulation of the antioxidant defense system, accumulation of proline, decline in MDA content, and increased concentration of chlorophyll and carotenoids were considered to be the protective effects of melatonin on the photosynthetic machinery of wheat to alleviate heat stress [90]. Furthermore, its application has been found to reduce oxidative damage in wheat by decreasing the levels of thiobarbituric acid reactive substances (TBARS) and H_2_O_2_ and enhancing photosynthetic efficiency through the activation of antioxidants [91]. Moreover, in rice leaves subjected to high temperatures, melatonin treatment resulted in enhanced antioxidant capacity, leading to an improvement in photosynthetic performance. The treated rice leaves maintained a fresh green appearance, and photosynthesis-related parameters with higher values, indicating better photosynthetic efficiency [92].

In tomato plants under heat stress conditions, the exogenous application of melatonin induces the expression of genes encoding the HSPs [93]. Notably, tomato plants exhibited a deceleration in heat-induced leaf senescence, as evidenced by reduced leaf yellowing, an elevated Fv/Fm ratio, and decreased ROS generation [94]. This effect was attributed to melatonin functioning to downregulate the expression of key genes involved in ROS production (e.g., *RBOHS*), genes associated with chlorophyll degradation and senescence. Furthermore, exogenous melatonin treatment in tomatoes leads to an increase in the endogenous levels of melatonin and gibberellins while simultaneously decreasing the levels of ABA [94]. In a study focusing on tomato photosynthesis under heat stress, researchers investigated the mechanism through which melatonin treatment improved photosynthesis when facing ROS production. The findings revealed that the melatonin treatment improved the net photosynthetic rate and chlorophyll fluorescence. Additionally, melatonin played a protective role in PSII by promoting a balanced electron transfer on the donor, reactive center, and acceptor sides, thus mitigating oxidative stress and damage to the photosynthetic machinery [95].

Cold or chilling stress also affects the photosynthetic machinery inducing photoinhibition at both PSII and PSI. However, melatonin treatment has shown promising results in mitigating these adverse effects in melons, rice, tomatoes, watermelons, and peppers.

In melons, melatonin-treated plants exhibited numerous positive responses to cold stress. The treatment led to an increased concentration of antioxidant enzymes, higher chlorophyll content, enhanced photosynthetic rate, improved stomatal conductance, and a higher maximal quantum yield of PSII. Moreover, the fluorescence parameters, especially the Fv/Fm ratio, significantly improved, indicating a reduction in photoinhibition. These findings also suggested that melatonin maintained chlorophyll stability in plants under cold stress, improving light-capturing efficiency and photosynthetic performance through the activation of RuBisCO [96]. Similarly, in rice seedlings subjected to cold stress, pretreatment with melatonin resulted in the improvement in photosynthesis parameters: higher net photosynthetic rate, increased stomatal conductance, elevated intercellular CO_2_, and enhanced water use efficiency [97]. When melatonin was applied to tomato seedlings, it yielded significant improvements in growth traits: pigment content, gas exchange elements, and chlorophyll fluorescence metrics [98]. Moreover, exogenous melatonin application also influences the expression of genes involved in phytohormone production in watermelons cultivated under cold-stress conditions. Key genes for jasmonic acid biosynthesis (Cl*AOC1*), and IAA biosynthesis (Cl*AMI1*) were upregulated in leaves due to melatonin treatment [99]. Finally, in *Capsicum annum* plants exposed to cold stress, melatonin treatment improved the photochemical activity of both PSII and PSI as well as the performance of essential photosynthetic enzymes, such as RuBisCO and fructose 1,6-biphosphatase. Furthermore, the exogenous supplementation of melatonin markedly improved photosynthetic pigment molecules, including Chlorophyll *a*, Chlorophyll *b*, and carotenoids. These improvement were attributed to the upregulation of pigments’ biosynthesis related-genes expression (Ca*CB12*, Ca*CAB4*, Ca*CAB7*, Ca*CAB8*, Ca*CAB21*, and Ca*CAB37*) [88].

In summary, melatonin treatment has been shown to exert positive effects on the photosynthetic performance of various crops under both heat and cold stress conditions. Table 3 provides a comprehensive overview of the physiological and biochemical changes occurring in the photosynthetic apparatus in response to melatonin treatment across different crops.

### 3.4. High Light Intensity

Despite the crucial importance of light in the photosynthesis process, the excess energy dissipated damages the photosystem complex, leading to photosynthetic efficiency decline. Exogenous melatonin is effective in alleviating cell membrane damage and inhibiting cell death under high-light conditions. In Arabidopsis thaliana, melatonin spray application enhanced tolerance to high light stress increasing chlorophyll and carotenoid contents, as well as the net photosynthetic rate ([100], Table 4). This protective effect of melatonin is primarily attributed to reducing the accumulation of ROS, preserving the integrity of membranes and photosynthetic pigments, and minimizing cell damage. To prevent harmful ROS levels, plants activate antioxidant enzymes, detoxification, and repair mechanisms [101].

### 3.5. Metal Toxicity

The productivity of crops worldwide faces a severe threat from heavy metal contamination including cadmium (Cd), copper (Cu), zinc (Zn), nickel (Ni), chromium (Cr), and others. Extensive research has focused on understanding the role of melatonin in regulating plant growth under various heavy metal stresses.

In tomato plants exposed to Cd stress, melatonin boosted H^+^-ATPase activity, and increased glutathione and phytochelatin concentrations, facilitating Cd sequestration within plant cells. This led to an improvement in the net photosynthetic rate and the maximum quantum efficiency of PSII photochemistry, resulting in ameliorative effects under Cd stress [102]. Similarly, the growth of strawberry seedlings cultivated in Cd-contaminated soil was severely hindered, leading to significantly lower growth rates, biomass output, chlorophyll levels, and antioxidant enzyme activities. However, the administration of melatonin demonstrated a remarkable increase in growth biomass output by improving the activity of antioxidant enzymes, such as APX, CAT, POD, and SOD, while reducing the accumulation of MDA [103].

The exogenous foliar application of melatonin alleviated copper toxicity in tomato plants via the upregulation of several defense genes’ expression (*CAT*, *APX*, *GR*, and *MDHAR*). This enhancement in gene expression indicated that melatonin plays a role in boosting antioxidant capacity and detoxifying Cu^2+^ by scavenging ROS, thereby alleviating copper toxicity [86]. Furthermore, in wheat plants subjected to nano-ZnO stress, melatonin spray application enhanced photosynthetic carbon assimilation; this was attributed to increasing RuBisCO and ATPase activity, along with higher chlorophyll concentration [104]. Melatonin administration has also shown promise in reducing Ni phytotoxicity in tomato seedlings. It enhanced gas exchange, increased nutrients, and photosynthetic pigment concentrations, as well as elevated the levels of minerals and secondary metabolites levels improving the net photosynthetic rate, transpiration rate, intercellular CO_2_ concentration, and stomatal conductance [105].

Melatonin has been found to be an effective component in reducing the harmful effects of Cr on canola and maize plant growth. By regulating photosynthesis, and improving the structural stability and efficiency of PSII and electron transport flow, it protects against photoinhibition due to oxidative damage [106]. Furthermore, the supplementation in maize plants resulted in a higher photosynthetic rate, increases in chlorophyll content, and improvement in antioxidant enzyme synthesis, effectively mitigating Cr toxicity. The research also explored the role of melatonin in regulating the heavy metal binding ability of the cell wall, further contributing to enhanced stress tolerance [107].

Table 5 summarizes a comprehensive overview of recent findings on the use of exogenous melatonin to reduce the impact of heavy metal stress in crop production.

### 3.6. UV Radiation

The rapid depletion of the ozone layer has resulted in the intensification of ultraviolet (UV) radiation, posing a serious threat to agricultural production. Natural sunlight reaching the Earth’s surface contains ultraviolet-B (UV-B) light, which can impact plant survival and adaptation. The plant’s response to UV-B light is influenced by several factors, such as the wavelength, intensity, and duration, as well as the presence of photosynthetically active radiation detected by phytochrome and cryptochrome photoreceptors.

**Figure 3:**
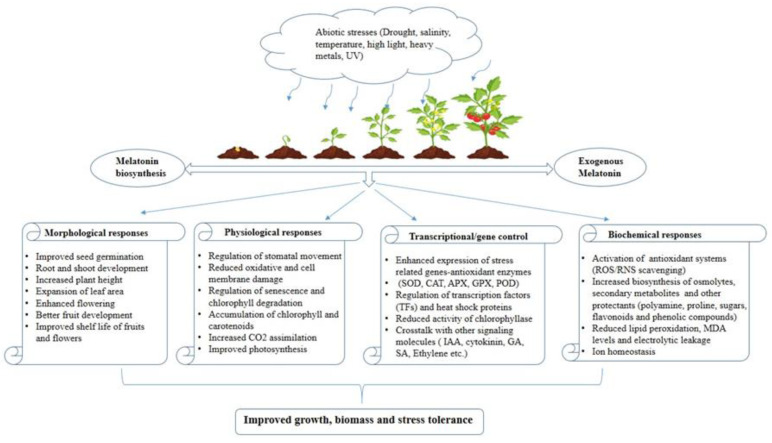
Response of plants treated with melatonin under abiotic stress conditions.

When melatonin was supplied, it effectively mitigated the inhibitory effects of UV-B radiation on various aspects of plant parameters. Notably, the detrimental impact on photosynthetic and chlorophyll fluorescence, stomatal apertures, chlorophyll levels, and leaf membrane damage were dramatically reduced. Additionally, melatonin exposure in the presence of UV-B stress led to an increase in the level of endogenous melatonin. The treatment is associated with the enhanced expression of the genes-encoding for the antioxidant enzymes APX and CAT which results in a reduction in the accumulation of H_2_O_2_ in *Arabidopsis* leaves exposed to UV-B radiation ([108]. Similar to this, melatonin-treated Arabidopsis subjected to UV-B stress demonstrated increased GR, APX, and SOD activity as well as lower lipid peroxidation levels and higher Fv/Fm values [109]. The melatonin application reduced the negative effects of UV-B stress on the biomass, photosynthetic pigment levels, and membrane lipids of the rosemary in vitro shoots increasing the activity of antioxidant enzymes and accumulation of total phenols ([110], Table 6). Figure 3 exhibits how plants generally react when exogenous melatonin is applied to them in a stressful environment.

## 4. Conclusion and Future Perspectives

Melatonin application has emerged as a promising strategy to enhance plant resistance against a wide array of abiotic stressors, encompassing drought, salinity, high/low temperature, UV radiation, high light, and heavy metal toxicity. The study of melatonin’s effects has garnered significant attention in the plant science field, especially with the discovery of the first receptor, CAND2/PMTR1, elevating melatonin’s status to that of a potential phytohormone. Numerous experiments have demonstrated that exogenous melatonin treatment leads to an increase in endogenous melatonin levels, enhancing plant resistance to abiotic stress. Exogenous melatonin showed outstanding resilience mechanisms in this context, including the regulation of plant growth, delay of leaf senescence, increasing photosynthesis, and enhancement of ROS and RNS scavenging antioxidant systems. Our comprehensive review underscores the multifaceted roles of exogenous melatonin in protecting the photosynthetic apparatus in plant systems under diverse abiotic stress conditions. These insights may help boost the production of environmentally friendly crops and ensure the safety of food.

However, despite the potential benefits of melatonin’s use in the horticulture sector, several challenges still need to be addressed, including standardization of optimal levels for effective application and the development of commercially viable sources of melatonin. As an alternative approach, earlier studies have explored the possibility of increasing endogenous melatonin synthesis within plants. For instance, transgenic *Arabidopsis* plants showed enhanced drought tolerance when the apple melatonin production gene Mz*ASMT1* was overexpressed [111]. Similarly, in *Arabidopsis*, ectopic overexpression of the grape melatonin synthesis gene Vv*SNAT1* resulted in the improvement in salt stress tolerance [112]. In rice, overexpressing the melatonin synthesis gene Os*COMT* led to a significant enhancement in the number and size of vascular bundles in culms and leaves [113]. Furthermore, another study demonstrated that under salt stress conditions, Mn*T5H2* actively promoted the conversion of tryptophan and tryptamine into melatonin, thereby, increasing its content and enhancing stress resistance in tobacco [114].

Despite significant advancements in understanding melatonin production genes, there is still a considerable amount to be explored regarding post-translational modifications and the regulatory mechanisms of associated proteins. To advance our understanding in this area, further research is essential and should be prioritized in the future. Melatonin serves as a pivotal regulatory component in the intricate network of phytohormones. Its interactions extend to various elements, including signaling molecules like NO, H_2_S, and ROS, as well as hormones such as auxin, ethylene, gibberellin, and abscisic acid. Unravelling these complex crosstalk interactions is crucial to comprehending the full scope of melatonin’s role in plant biology, particularly under stress conditions. To shed light on the interactions between melatonin and these signaling molecules, future research must employ innovative approaches. Creating similar mutants through gene editing and plastid transformation techniques offers a promising avenue to investigate the responses of plants to stressed environments. By utilizing these mutant plants, we can better dissect the intricate regulatory pathways through which melatonin influences plant responses to abiotic stress.

The insights gained from such research endeavors will not only enhance our knowledge of melatonin’s multifaceted role in stress tolerance but also hold great potential for developing strategies to improve crop resilience in the face of environmental challenges. Therefore, investing in future research on melatonin’s crosstalk with signaling molecules and hormones is of the utmost importance for sustainable agriculture and food security.

## Figures and Tables

**Table 1:** Effects of exogenous melatonin application on photosynthetic apparatus under drought stress.

Stress	Crop	Effect on Photosynthesis	Reference
Drought	Maize	Improvement of the number, length, and width of stomata, and stabilization of the chloroplast structure, increasing photosynthesis.	[62]
	Ranunculus asiaticus	Accumulation of chlorophyll and carotenoids.	[75]
	Phoebe	Increase photosynthetic efficiency by enhancing the concentration of photosynthetic pigment and regulation of phytohormones such as jasmonic acid and ABA, scavenging ROS.	[76]
	Chrysanthemum	Improvement of photosynthetic efficiency reduces the loss of relative water content and chlorophyll in leaves, regulation of osmotic metabolism by increasing the concentration of soluble proteins and sugars.	[73]
	Maize	Enhances the stomatal length, width, area, and number of pores. Mitigates ROS-induced oxidative damages by increasing the photosynthetic pigments, antioxidant enzyme activities, relative water content, and osmoprotectants.	[61]
	Moringa	Enhances the photosynthetic pigments, phenolic contents, and IAA accumulation. Reduction of ROS accumulation by increasing the activities of APX, CAT, and SOD.	[74]
	Coffee	Enhances gas exchange, improves carboxylation efficiency, and chlorophyll content, and reduces damage to the photosynthetic machinery.	[63]
	Triticale	Increases stomatal conductance, net photosynthetic rate, transpiration rate and chlorophyll concentration.	[71]

**Table 2:** Effects of exogenous melatonin application on photosynthetic apparatus under salt stress.

Stress	Crop	Effects on Photosynthesis	Reference
Salinity	Wheat	High water status, low level of H_2_O_2_ content, and balanced [K^+^]/[Na^+^] ratio in leaves, high content of proline, protein, soluble sugars essential amino acids and Ca^2+^ in leaves resulting in a high photosynthetic rate.	[86]
	Sugarbeet	Increases the level of photosynthetic net rate, chlorophyll fluorescence, and chlorophyll content.	[81]
	Cotton	Reduction of ROS production, increases plant biomass and chlorophylls level, preservation of mitochondria and grana lamella of chloroplasts.	[84]
	Rice	Upregulation of gene expression associated with antioxidant system, photosynthetic parameters, and ROS scavenging enzymes. Reduction of electrolyte leakage.	[83]
	Tomato	Reduction of chlorophyll degradation; alleviated PSII inhibition and OEC damage.	[78]
	Oats	Upregulation of genes encoding ROS scavenging enzymes, stress-responsive, NAC transcription factors, and PSII core proteins. Increases stomatal conductance and chlorophyll content. Reduced the levels of MDA and electrolyte leakage.	[13]
	Cucumber	Improvement of photosynthetic efficiency, inhibition of chlorophyll degradation, reduction of MDA and ROS contents. Increased the expression of antioxidant-associated genes.	[77]

**Table 3:** Effects of exogenous melatonin application on photosynthetic apparatus under temperature stress.

Stress	Crop	Effects on Photosynthesis	Reference
Heat stress	Tomato	Improves photosynthetic parameters, such as net photosynthetic rate and chlorophyll fluorescence. Reduces oxidative stress to PSII.	[95]
	Rice	Enhances the photosynthetic capacity. Promotes the fresh green appearance of leaves and higher values of photosynthesis-related parameters.	[92]
	Wheat	Reduces oxidative damage by lowering the TBARS and H_2_O_2_ content. Improves photosynthetic efficacy through enhancement of antioxidants. Improves photosynthetic efficacy through enhancement of antioxidants, accumulation of proline, chlorophyll and carotenoids.	[90,91]
	Tomato	Increases Fv/Fm ratio and chlorophyll levels. Decreases ROS generation. Reduces expression of *RBOHS* and chlorophyll degradation-associated genes.	[94]
Cold stress	Melon	Increases concentration of antioxidant enzymes, chlorophyll content, photosynthetic rate, stomatal conductance, and maximal quantum yield of PS II.	[96]
	Rice	Enhances net photosynthetic rate, stomatal conductance, intercellular CO_2_ and water use efficiency.	[97]
	Tomato	Improves pigment content, gas exchange elements, and chlorophyll fluorescence metrics.	[98]
	Pepper	Improves photochemical activity of PSII and PSI and photosynthetic enzymes. Increases the levels of chlorophyll *a*, chlorophyll *b*, and carotenoids. Enhances photosynthesis under cold stress conditions.	[88]

**Table 4:** Effects of exogenous melatonin application on photosynthetic apparatus under high light stress.

Stress	Crop	Effects on Photosynthesis	Reference
High light	*Arabidopsis*	Increases photosynthetic rate, chlorophyll content and reduces ROS levels.	[100]

**Table 5:** Effects of exogenous melatonin application on photosynthetic apparatus under heavy metal stress.

Stress	Crop	Effects on Photosynthesis	Reference
Cr toxicity	Maize	Improves photosynthetic rate, chlorophyll content, and antioxidant enzyme synthesis.	[107]
Cr toxicity	Canola	Prevents photo-inhibition of PSII from oxidative damage.	[106]
Zn toxicity	Wheat	Increases photosynthetic carbon assimilation, RUBISCO and ATPase activity, and chlorophyll concentration.	[104]
Cd toxicity	Tomato	Increases in Fv/Fm ratio and net photosynthetic rate increase activity of H+-ATPase activity, and reduces ROS accumulation.	[102]
Cu toxicity	Tomato	Upregulate antioxidant-associated gene expression.	[86]
Ni toxicity	Tomato	Improves photosynthetic and transpiration rate, intercellular CO_2_ concentration, and stomatal conductance.	[105]
Cd toxicity	Strawberry	Increases activity of antioxidant enzymes.	[103]
Cr toxicity	Maize	Improves photosynthetic rate, chlorophyll content, and antioxidant enzyme synthesis.	[107]

**Table 6:** Effects of exogenous melatonin application on photosynthetic apparatus under UV stress.

Stress	Crop	Effects on Photosynthesis	Reference
UV radiation	*Arabidopsis*	Upregulated the genes encoding for ascorbate peroxidase and catalase, lowered lipid peroxidation and increased the Fv/Fm value	[108,109]
	Rosemay	Increased the activity of SOD, CAT and POD, and the accumulation of total phenols	[110]
